# Editorial 'Men's Health'

**DOI:** 10.1080/2090598X.2021.1968135

**Published:** 2021-09-16

**Authors:** Ashok Agarwal, Ahmad Majzoub, Mohamed Arafa, Haitham ElBardisi

**Affiliations:** American Center for Reproductive Medicine Director, Andrology Laboratory, Lerner College of Medicine, Cleveland Clinic, Cleveland, Ohio, USA, agarwaa@ccf.org; Urology, Andrology and Male Infertility Department of Urology, Hamad Medical Corporation, Doha, Qatar; Clinical Urology, Weill Cornell Medicine- Qatar, Doha, Qatar

Interest in gender-based medicine began in the 1960s with the emergence of the Women’s Health Movement in the USA[1]. Whereas, the interest in research on Male health started a decade later and coincided with advancements of the urology specialty[2]. However, advances in this field went on a slow pace till early 2000 when the study of men’s health witnessed major breakthroughs, allowing urologists to implement preventive and early therapeutic measures for a wide range of male health conditions. Some of these advances were: 1) in the field of male infertility, the development of different sperm retrieval methods allowed azoospermic patients who were otherwise deemed sterile, to father their biologic children[3]. Advancements in testicular tissue handling and sperm processing enhanced the surgical sperm retrieval rate and improved the outcome of assisted reproductive therapies in these patients. 2) improved understanding of the etiology and pathophysiology of premature ejaculation allowed the discovery of new treatment modalities that were solely approved to treat such condition[4], 3) erectile dysfunction related research resulted in new treatments such as low intensity shock wave therapy and refinement in prosthetic surgery[5], 3) advances in testosterone replacement therapy ignited the importance of testosterone in various bodily functions other than sexual and reproductive health, resulting in improvement in the life style of males with hypogonadism[6],and finally, 4) recent interest in chronic pelvic pain syndrome, a debilitating clinical condition, allowed the institution of new methods for patient evaluation and the adaptation of a multidisciplinary approach to treatment which was proven to be effective in relieving patient suffering [7][].

The aim of this special issue is to shed light on key aspects of men’s health and to explore the latest advancements in this field of medicine. Section one discusses male reproduction focusing mainly on azoospermia with a series of articles that cover evaluation, medical treatment, surgical sperm retrieval and tissue and sperm handling following surgery. Section two focuses on sexual health and covers topics such as premature ejaculation, peyronies’ disease and erectile dysfunction. Section three focuses on chronic pelvic pain syndrome and explores new methods for patient evaluation and treatment. Finally, section four handles sexually transmitted diseases as well as emerging conditions and their implication on male sexual and reproductive health.

We are confident that this special issue will be a useful guide for urologists, gynecologists, andrologists, embryologists, and other healthcare workers practicing reproductive medicine. In addition, it will be a valuable resource for students and researchers wishing to learn more about this subject. We are grateful to the team of highly acclaimed contributors, who have worked hard to share their latest, thoroughly researched and well-written articles. This publication would not be possible without their active support. The authors wish to thank Professor Ahmed Shokeir, Editor-in-Chief of the Arab Journal of Urology for his invitation to produce a special issue focused on Men’s Health. We are indebted for his guidance and support at each stage of development. Lastly, we are grateful to the thousands of our patients for the privilege to help them.
